# Muscle Carnitine Palmitoyltransferase II Deficiency: A Review of Enzymatic Controversy and Clinical Features

**DOI:** 10.3390/ijms18010082

**Published:** 2017-01-03

**Authors:** Diana Lehmann, Leila Motlagh, Dina Robaa, Stephan Zierz

**Affiliations:** 1 Department of Neurology, Martin-Luther-University Halle-Wittenberg, Ernst-Grube-Str. 40, 06120 Halle/Saale, Germany; leila.motlagh@medizin.uni-halle.de (L.M.); stephan.zierz@uk-halle.de (S.Z.); 2 Institute of Pharmacy, Martin Luther University Halle-Wittenberg, Halle (Saale), Wolfgang-Langenbeck-Str. 4, 06120 Halle/Saale, Germany; dina.robaa@pharmazie.uni-halle.de

**Keywords:** carnitine palmitoyltransferase, myoglobinuria, myopathy, muscle, CPT (carnitine palmitoyltransferase) II deficiency, enzyme activity, enzyme structure

## Abstract

CPT (carnitine palmitoyltransferase) II muscle deficiency is the most common form of muscle fatty acid metabolism disorders. In contrast to carnitine deficiency, it is clinically characterized by attacks of myalgia and rhabdomyolysis without persistent muscle weakness and lipid accumulation in muscle fibers. The biochemical consequences of the disease-causing mutations are still discussed controversially. CPT activity in muscles of patients with CPT II deficiency ranged from not detectable to reduced to normal. Based on the observation that in patients, total CPT is completely inhibited by malony-CoA, a deficiency of malonyl-CoA-insensitive CPT II has been suggested. In contrast, it has also been shown that in muscle CPT II deficiency, CPT II protein is present in normal concentrations with normal enzymatic activity. However, CPT II in patients is abnormally sensitive to inhibition by malonyl-CoA, Triton X-100 and fatty acid metabolites. A recent study on human recombinant CPT II enzymes (His_6_-*N*-*h*CPT2 and His_6_-*N*-*h*CPT2/S113L) revealed that the wild-type and the S113L variants showed the same enzymatic activity. However, the mutated enzyme showed an abnormal thermal destabilization at 40 and 45 °C and an abnormal sensitivity to inhibition by malony-CoA. The thermolability of the mutant enzyme might explain why symptoms in muscle CPT II deficiency mainly occur during prolonged exercise, infections and exposure to cold. In addition, the abnormally regulated enzyme might be mostly inhibited when the fatty acid metabolism is stressed.

## 1. Introduction

The carnitine palmitoyltransferase (CPT) system consists of two enzymes, CPT I and CPT II, and is involved in the transport of long-chain fatty acids into the mitochondrial compartment. The enzymes are located in the outer (CPT I) and inner mitochondrial membrane (CPT II). Three phenotypes of CPT II deficiency are known: a lethal neonatal form, a severe infantile hepatocardiomuscular form, and a mild myopathic form [1]. Muscle CPT II deficiency is the most frequent type of CPT II deficiency. The disease follows an autosomal recessive mode of inheritance. In approximately 90% the molecular basis is a p. S113L mutation in homozygous or heterozygous state with an allele frequency of 60%–70% [2,3,4]. In addition there are more than 60 mostly private mutations [4].

Clinical features are attacks of muscle weakness, myalgia, pain and rhabdomyolysis with or without renal failure. Trigger factors are prolonged exercise, fasting, fever and exposure to cold [5].

The biochemical consequences of the disease-causing mutations are still discussed controversially. In former studies, CPT activities in muscles of patients with CPT II deficiency ranged from not detectable [6,7,8,9] to reduced [10,11,12,13,14] up to normal [15,16]. CPT I but not CPT II is sensitive to inhibition by malonyl-CoA. Trevisan et al. showed an almost complete inhibition of total CPT activity in patients by malonyl-CoA [17]. From this it was inferred that the normal malonyl-CoA–insensitive CPT II activity is deficient. However, it has also been shown that total CPT activity is normal under optimal assay conditions but abnormal when inhibited by malonyl-CoA, palmitoylcarnitine, carnitine and Trition-X100 (non-ionic surfactant). This led to the hypothesis of an abnormally regulated enzyme with a normal total CPT II concentration [15,16,18]. Zierz et al. [19] showed that CPT II muscle deficiency patients have an enzymatically active CPT II which is abnormally sensitive to inhibition by Tween (nonionic detergent), and that CPT I activity is not compensatorily increased in these patients. However, after preincubation of the muscle homogenate of CPT II muscle deficiency patients with trypsin, the total CPT activity slightly increased and rendered the activity greatly insensitive to inhibition by malonyl-CoA in both patients and controls [20]. In one Western blot study on one patient, there was no detectable CPT II protein at all [21]. In another immunoreactivity study, prior to the identification of the disease-causing mutations, five groups of patients were differentiated according to enzyme activity and protein content, but none of the patients had complete loss of the CPT protein [16]. However, in these studies, antibodies against bovine liver CPT II [21] and rat liver CPT II [16] have been used. The p.S113L mutation represents a missense mutation, and does not lead to a truncated protein [22]. This argues against a complete loss of CPT II protein. From transfection experiments of COS (CV-1 in Origin, carrying SV40) cells with the p.S113L mutation, a normal synthesis but markedly reduced steady-state level of the protein was postulated [22]. In a study, fibroblast cultures preincubated for three weeks at 37 and 41 °C and the subsequent measurement of fatty acid oxidation at 37 and 41 °C, respectively, showed reduced fatty acid oxidation in patients [23]. From this, thermal instability of the mutant enzyme has been postulated [23]. This hypothesis could be confirmed in a recent study using human recombinant CPT II enzymes. In this study, the wild-type and the variant S113L showed the same enzymatic activity. However, the mutant enzyme showed a marked thermolability and was also abnormally inhibited by malonyl-CoA [24,25].

## 2. Clinical Presentation of Patients with Muscle CPT II

Three different phenotypes of CPT II deficiency are known: the multisystemic lethal neonatal, the infantile and the adult myopathic forms. In contrast to CPT I, there are no tissue-specific isoforms of CPT II. Thus, the clinical heterogeneity of CPT II deficiency is due to different mutations. Joshi et al. (2014) [5] analyzed a cohort of 50 patients’ muscle CPT II deficiency retrospectively. Thirty-two patients included in that study have already been described previously [2,24,26]. Sixty percent of the patients had an early childhood onset compared to later adolescent or adulthood onsets. Almost all patients (94%) described attacks of myalgia. Following the main clinical symptoms were myoglobinuria (86%) and muscle weakness (76%) [5]. The most common trigger factors were exercise (87%) and infection (62%). The diagnosis can be confirmed by molecular investigations. In 90% of the patients, the S113L mutation has been found on the CPT II gene, with an allele frequency of 60%–70% [2,3,4].

## 3. Biochemical Studies in Patients with CPT II Muscle Deficiency

In a previous study on muscle biopsies of nine patients with genetically proven CPT II deficiency, the enzyme was investigated. The genotypes were p.S113L/p.S113L (*n* = 4), p.S113L/p.R231W (*n* = 1), p.S113L/p.Y479F (*n* = 1), p.S113L/c.1646_1649del (*n* = 1), p.S113L/c.1238_1239del (*n* = 1), and p.S113L/p.P50H (*n* = 1) [25]. Total CPT activity of patients in the isotope forward assay was not significantly different from that of controls. The remaining activities upon inhibition by malonyl-CoA and Triton X-100 were only 25% of those in controls [25]. Immunohistochemically, CPT II could be demonstrated with the same intensity in patients as in controls. In Western blot studies, COX (cytochrome *c* oxidase) was used as a mitochondrial marker for the quantification of CPT II protein. Patients and controls all showed the same staining intensity [25].

## 4. Thermolability of the S113L Variant

**Figure 1:**
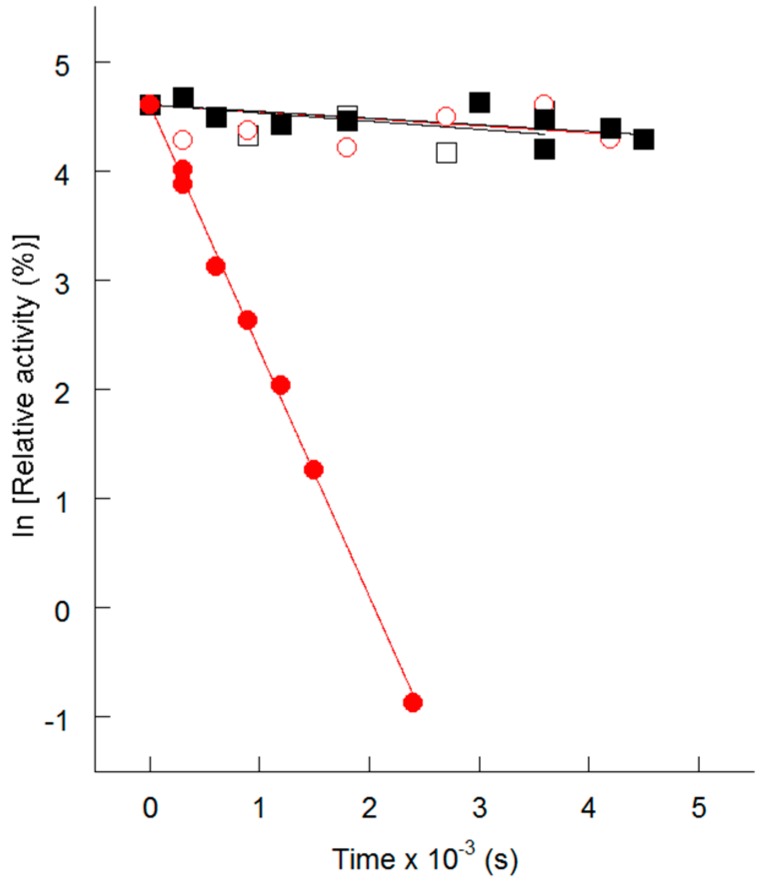
Thermal inactivation of His_6_-*N*-*h*CPT2 (open symbols) and His_6_-*N*-*h*CPT2/S113L (filled symbols) at 30 and 40 °C. Black squares show thermal inactivation at 30 °C, red circles represent values at 40 °C. The data is presented as time-dependent changes of natural-log-transformed relative activities.

His_6_-*N*-*h*CPT2 (wild type) and His_6_-*N*-*h*CPT2/S113L (variant) were expressed recombinantly in prokaryotic hosts. The enzyme activity was determined spectroscopically according to Rufer et al. [27] with some modifications [28,29]. Temperature-induced inactivation of CPT II was analyzed after incubation of the enzymes at 40 and 45 °C. The results showed a significantly faster decrease of the enzyme activity of the mutated enzyme compared to the wild type at both temperatures (40 and 45 °C) (Figure 1) [29]. A recent study supported the findings of thermolability in CPT II deficiency [30]. Cultured fibroblasts of three types of CPT II variants (p.V368I (heterozygous); p.V368I (homozygous); p.F352C (heterozygous) + p.V368I (homozygous)) showed decreased enzyme activities, cellular β-oxidation and ATP generation. The *Km* value for l-carnitine, thermal instability, short half-lives, and cellular apoptosis were increased [30]. In order to study the effect of the S113L mutation on the thermostability of the enzyme, molecular dynamics (MD) simulations were performed on the wild-type and mutant enzymes at different temperatures using a generated homology model of human CPT II. These simulations confirmed the thermolability of the S113L variant. The calculated B-factor (indicating the flexibility of the backbone) for the residues neighboring the mutation site (S110-L121) showed a significantly higher fluctuation for the mutant’s residues at 313 K (40 °C) when compared to 277 K (4 °C), and to a lesser extent at 293 K (20 °C). In contrast, the calculated B-factors for the wild-type enzyme revealed no noticeable differences at the three above-mentioned temperatures [29].

## 5. Protective Effect of Natural Substrates

Motlagh et al. studied a putative substrate protection effect on the kinetic stability of the enzymes [29]. After their pre-incubation with various natural substrates at different temperatures, the kinetic stability of the enzymes was measured [29].

Pre-incubation of the recombinant wild-type S113L variant with the native substrate palmitoyl-CoA prior to the addition of l-carnitine revealed no substrate protection and generally increased the rate of thermal inactivation at 40 and 45 °C. In contrast, both enzymes displayed a much higher kinetic stability on pre-incubation with l-carnitine at 45 °C [29].

The middle-chain acyl-l-carnitines, C10, C12 and C14, and the long-chain one, C16, stabilized the mutated enzyme to the level of the wild-type at 45 °C. At 40 °C they could decrease the inactivation rate constant of the wild-type and the variant S113L by a factor of about 1000 and 25, respectively [29]. MD studies on the wild-type and the variant S113L in complex with palmitoyl-l-carnitine showed no differences in the behavior of both enzymes with increasing temperature, indicating the stabilization effect of palmitoyl-l-carnitine on the S113L variant. The calculated B-factor of the residues surrounding the mutation site (S110-L121) in the complex did not show any increase at higher temperatures (313 K, 40 °C). Generally, a lower flexibility of the acyl-l-carnitine binding site residues as well as of the whole protein was observed for the variant S113L in complex with palmitoyl-l-carnitine compared to the protein without substrate at 40 °C [29].

## 6. Inhibitory Effect of Malonyl-CoA on CPT II

**Figure 2:**
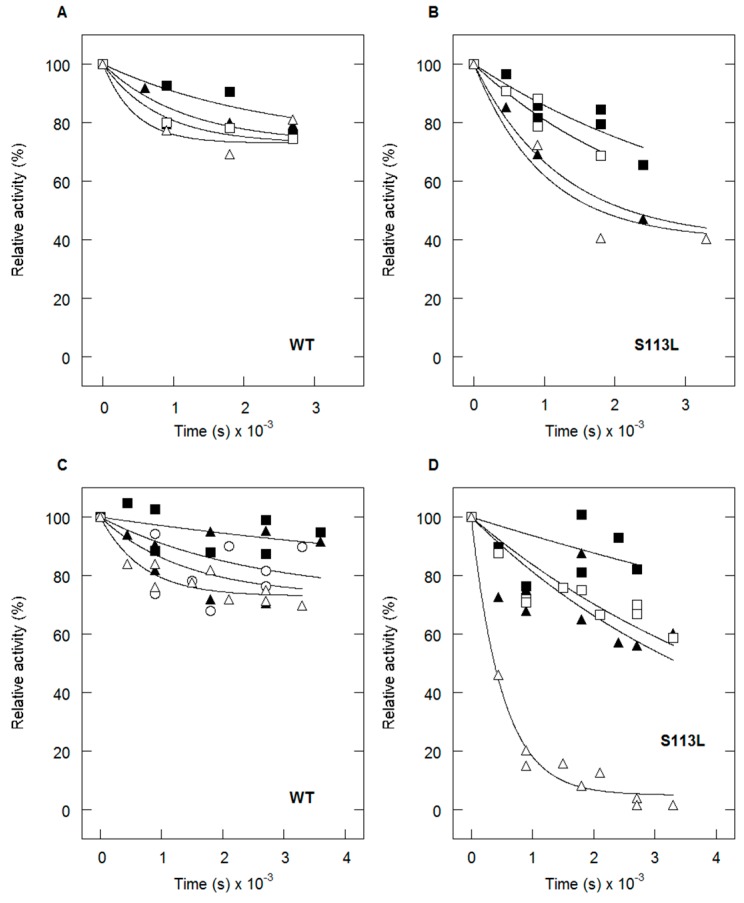
Effect of malonyl-CoA and malonic acid (malonate) on the kinetic stability of recombinant CPT II enzymes. Inactivation of His_6_-*N*-*h*CPT2 at different concentrations (squares: 10 µM, triangles: 200 µM inhibitor) and temperatures (closed symbols: activity at 4 °C, open symbols: activity at 30 °C). (**A**) by malonyl-CoA and (**C**) by malonic acid (malonate). Inactivation of His_6_-*N*-*h*CPT2/S113L at different concentrations and temperatures (**B**) by malonyl-CoA and (**D**) by malonic acid (malonate). The data is shown as time-dependent change of relative activities.

Previously, it has been shown that in muscle homogenates of patients with CPT II deficiency, limited trypsin proteolysis rendered total enzyme activity (i.e., CPT I and II) almost completely insensitive to inhibition by malonyl-CoA [20]. Motlagh et al. evaluated the inhibitory effect of malonyl-CoA and malonic acid (malonate) on CPT II [28]. The activities of His_6_-*N*-*h*CPT2 and His_6_-*N*-*h*CPT2/S113L were measured by pre-incubation of these effectors at three different concentrations (10, 100 or 200 µM) (Figure 2). A time-dependent inhibitory effect of the metabolites has been shown. While the wild-type displayed a residual final activity of about 70% in the presence of malonyl-CoA, the S113L variant decreased to 40%. Pre-incubation of the enzymes with malonic acid resulted in a residual activity of about 70% in the wild-type but of about 5% in the variant.

**Figure 3:**
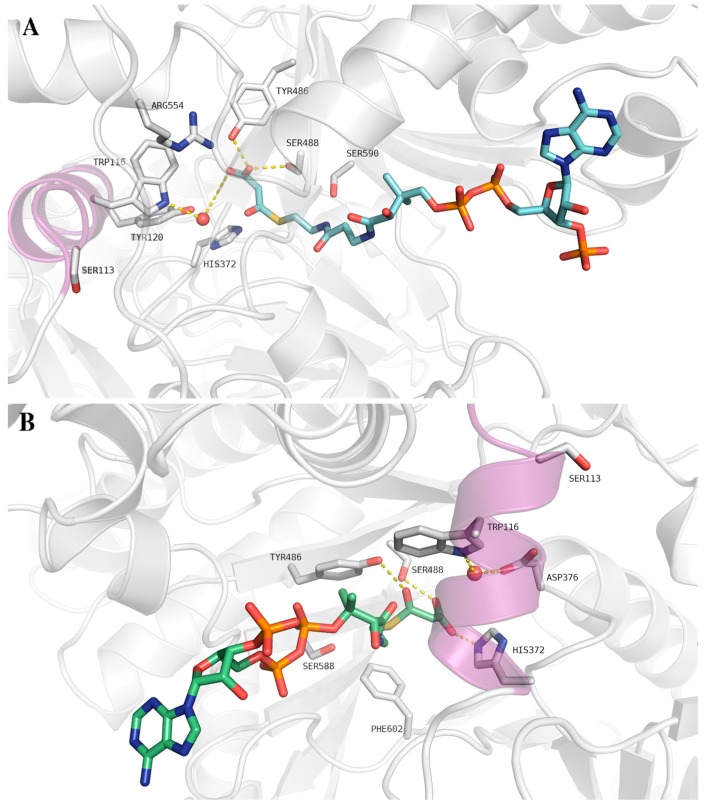
Docking studies of malonyl-CoA with CPT II. (**A**) Interaction of malonyl-CoA (cyan) docked to site I of CPT II; (**B**) Interaction of malonyl-CoA (cyan) docked to site II of CPT II. The conserved water molecule W88 is shown as a red sphere. The α-helix bearing the S113L mutation is shown as a magenta ribbon. Only residues of the catalytic site are shown as white sticks for clarity. Hydrogen bonds are shown as yellow dashed lines.

Docking studies using the homology model of human CPT II revealed two different binding sites for malonyl-CoA and malonic acid (malonate) [28] (Figure 3).

By addition of the native substrate palmitoyl-CoA and without the other substrate carnitine, the activity of the native enzyme was restored to normal wild-type levels only 60 s after starting the enzyme assay (post-incubation). However, the residual activity of the variant S113L could not been restored [28].

A conceivable reason behind the abnormal inhibition of the S113L CPT II variant could be deduced from the obtained docking results (Figure 3). Although the Ser113 residue is not directly located in the binding pocket, its mutation to the leucine hydrophobic residue might lead to a change in the conformation of the binding pocket, altering the location of catalytically important residues. The suggested conformational change induced by the S113L mutation could either lead to an enhancement of the binding of malonyl-CoA or malonate, or result in a weaker binding of the native substrate. Thus, the native substrate cannot efficiently compete with the tightly bound malonyl-CoA or malonate. This could also explain why the enzymatic activity of the S113L variant is only partly restored by post-incubation with palmitoyl CoA [28].

Malonyl-CoA is synthesized by acetyl-CoA carboxylase (ACC). There are two isoforms of ACC: (i) ACC1 mainly localized in lipogenic tissues such as the liver and adipose tissue; and (ii) ACC2 present in the heart and skeletal muscle but also in the liver [31]. Malonyl-CoA is found in the liver, heart and skeletal muscle [32]. In the rat liver, the malonyl-CoA content is high in the fed state and decreased during fasting, exercise, and in diabetes [33,34]. In skeletal muscle, the inhibitory constant (I_50_) of CPT I for malonyl-CoA is only 13%–23% of that in the liver, indicating a higher sensitivity of CPT I for malonyl-CoA inhibition in skeletal muscle compared to the liver [34]. It has been suggested that malonyl-CoA contributes to the regulation of de novo fatty acid synthesis by inhibiting fatty acid synthesis in the fed state. During fasting, the decreased malonyl-CoA concentration might facilitate mitochondrial fatty acid utilization. The physiological significance of the slight inhibition of normal CPT II by malonyl-CoA has not been established. However, due to the abnormally high sensitivity of the mutant CPT II for malonyl-CoA, it can be speculated that even the reduced malonyl-CoA level during fasting is still sufficient to significantly inhibit CPT II activity in patients with CPT II deficiency. This in turn might contribute to triggering symptoms in patients during fasting and prolonged exercise whereas the wild-type CPT2 is not affected.

## 7. Summary and Conclusions

In previous studies, muscle carnitine palmitoyl transferase II deficiency was mostly considered to be associated with adult or late onset [22,35,36,37] rather than early childhood manifestation [38,39,40,41,42,43]. However, Joshi et al. (2014) [5] showed that the manifestation of clinical symptoms occurred more frequently during infancy (one to 12 years old) than during adolescence (13–22 years old) and adulthood (>22 years old). The main clinical symptoms in patients with muscle carnitine palmitoyl transferase II deficiency are attacks of myalgia and myoglobinuria, possibly leading to renal failure. Infections and exposure to cold seem to be the most common trigger factors.

In muscle CPT II deficiency, symptoms occur only intermittently. This is in contrast to carnitine deficiency [44]. The normal protein content and enzyme activity allow a normal function of the CPT system in situations without stress on the fatty acid metabolism [25,29]. CPT II with the S113L mutation, however, is most vulnerable to inhibition when it is most needed [29].
